# Short- and long-term outcomes of surgery for colorectal and non-colorectal liver metastasis: a report from a single center in the Baltic country

**DOI:** 10.1186/s12957-020-01944-2

**Published:** 2020-07-14

**Authors:** Rokas Račkauskas, Augustinas Baušys, Vitalijus Sokolovas, Marius Paškonis, Kęstutis Strupas

**Affiliations:** Clinic of Gastroenterology, Nephrourology, and Surgery, Institute of Clinical Medicine, Faculty of Medicine, Vilnius University, Ciurlionio str. 21, 03101 Vilnius, Lithuania

## Abstract

**Background:**

The liver is a major target organ for metastases of various types of cancers. Surgery is a well-established option for colorectal liver metastases (CRLM). Regarding the improved surgical and anesthetic techniques, the safety of liver resection has increased. Consequently, the interest in the surgical management of non-colorectal liver metastases (non-CRLM) has gained significant attention. Therefore, this study was designed to investigate the surgical treatment outcomes for non-CRLM and to compare it with an outcome of CRLM in a tertiary care center in the Baltic country—Lithuania.

**Methods:**

We retrospectively analyzed data from all patients who underwent liver resection for CRLM or non-CRLM between 2010 and 2017 in a tertiary care center—Vilnius University hospital Santaros Clinics. Demographic and metastasis characteristics, as well as disease-free and overall survival, were compared between the study groups.

**Results:**

In total, 149 patients were included in the study. Patients in the CRLM group were older (63.2 ± 1.01 vs 54.1 ± 1.8 years, *p* < 0.001) and mainly predominant by males. Overall postoperative morbidity rate (16.3% vs 9.8%, *p* = 0.402) and major complications rate (10% vs 7.8%, *p* = 0.704) after liver resection for CRLM and non-CRLM was similar. Kaplan-Meier analysis showed higher disease-free survival in the CRLM group with 89.4% vs 76.5% and 64.9% vs 31.4% survival rates at 1 and 3 years, respectively (*p* = 0.042), although overall survival was not different between the CRLM and non-CRLM groups with 89.4% vs 78.4% and 72.0% vs 46.1% survival rates at 1 and 3 years, respectively (*p* = 0.300).

**Conclusions:**

In this study, we confirmed comparable short- and long-term outcomes after liver resection for CRLM and non-CRLM. Surgical resection should be encouraged as an option in well-selected patients with non-CRLM.

## Background

The liver is a major target organ for metastases of various types of cancer. Colorectal cancer (CRC) is one of the most common cancers worldwide [1, 2] and about 50% of CRC patients will develop colorectal liver metastases (CRLM) throughout their course of disease [3, 4]. Surgery for CRLM is well defined by current guidelines, and it is the only treatment method that may be potentially curative. In contrast, the therapeutic approaches for non-colorectal liver metastases (non-CRLM) remain controversial. Due to the wide heterogeneity of origin, the different biological behavior of different cancers, and the relatively lower incidence, there are no strict guidelines on how to manage non-CRLM. Systemic treatment for non-CRLM is available, although it does not offer satisfactory results, with survival for only a few months [5]. Regarding, the improved surgical and anesthetic techniques, the safety of liver resection has improved over the last decades [5]. Consequently, the interest in the surgical management of non-CRLM has gained significant attention, although the surgery for non-CRLM remains non-standardized. Thus, there is a need for studies investigating short- and long-term outcomes after liver resection for non-CRLM. Moreover, the outcomes of liver surgery strongly depend on a surgeon and a hospital volume [6]. While the centralization of liver surgery in large and well-developed countries is feasible, it may remain challenging in smaller or developing countries. Therefore, this study was designed to investigate the outcomes of surgery for non-CRLM and to compare it with outcomes after liver resection for CRLM in a tertiary care center in the Baltic country—Lithuania.

## Materials and methods

### Ethics

Vilnius regional biomedical research ethics committee approval (No. 158200-18/7-1054-553) was obtained before this study was conducted. The study was conducted according to the principles of the Declaration of Helsinki.

### Inclusion criteria

All patients who underwent liver resection for CRLM or non-CRLM between January 1, 2010, and December 31, 2017, at a tertiary care center—Vilnius University hospital Santaros Clinics—were included in the study. In all cases, surgical resection was performed after patients were discussed at a multidisciplinary tumor board.

### Data collection

Data on patient characteristics were extracted from the prospectively collected institutional electronic database. They included age; gender; the history of previous cancer treatment; origin, number, and size of metastases; surgical approach (open surgery, laparoscopic surgery); and intraoperative data such as length of surgery, blood loss, and postoperative complications by Clavien-Dindo classification.

### Study outcomes

The primary outcome of the study was overall survival (OS). The secondary outcomes included disease-free survival (DFS) and postoperative morbidity. OS was defined as the time from liver resection to death. Data on survival and date of death were collected from the National Lithuanian Cancer registry. DFS was defined as the time from surgery to disease progression including local or distant recurrence.

### Statistical analysis

All statistical analyses were conducted using the statistical program SPSS 24.0 (SPSS, Chicago, IL, USA). Continuous variables are presented as the mean ± standard deviation or median with an interquartile range where appropriate. Categorical variables are shown as proportions. Continuous variables were compared by a *t* test or ANOVA, and categorical variables by the Pearson’s chi-square test. Overall and recurrence-free survival rates were analyzed by the Kaplan-Meier method and compared by the log-rank test. Statistical significance was considered when *p* value < 0.05 was achieved.

## Results

### Baseline characteristics

In total, 149 patients were included in the study. Based on the origin of the metastases, 98 (65.7%) were allocated to CRLM and 51 (34.2%) to the non-CRLM group. The baseline clinicopathological characteristics are shown in Table 1. Patients in the CRLM group were older (63.2 ± 1.01 vs 54.1 ± 1.8 years, *p* < 0.001) and mainly predominant by males, while in the non-CRLM group by females (60/38 vs 11/40, *p* < 0.001).

**Table 1:** Baseline clinicopathologic characteristics in patients with colorectal liver metastasis and non-colorectal liver metastasis

	CRLM (***n*** = 98)	non-CRLM (***n*** = 51)	***p*** value
Gender (males/females)	60/38	11/40	< 0.001
Age, years (mean ± SD)	63.2 ± 1.01	54.1 ± 1.8	< 0.001
Length of stay, days (mean ± SD)	9.3 ± 0.4	8.4 ± 0.3	0.146
CA125, U/ml (mean ± SD)		2168.7 ± 1272.3	N/A
CEA, ng/l (mean ± SD)	150.5 ± 69.1	15.2 ± 7.2	0.998
CA 19.9 ng/l (mean ± SD)	325.0 ± 75.9	14316.1 ± 11919.77	0.063

*CRLM* colorectal liver metastases, *non-CRLM* non-colorectal liver metastases, *SD* standard deviation

### Liver metastasis

**Fig. 1:**
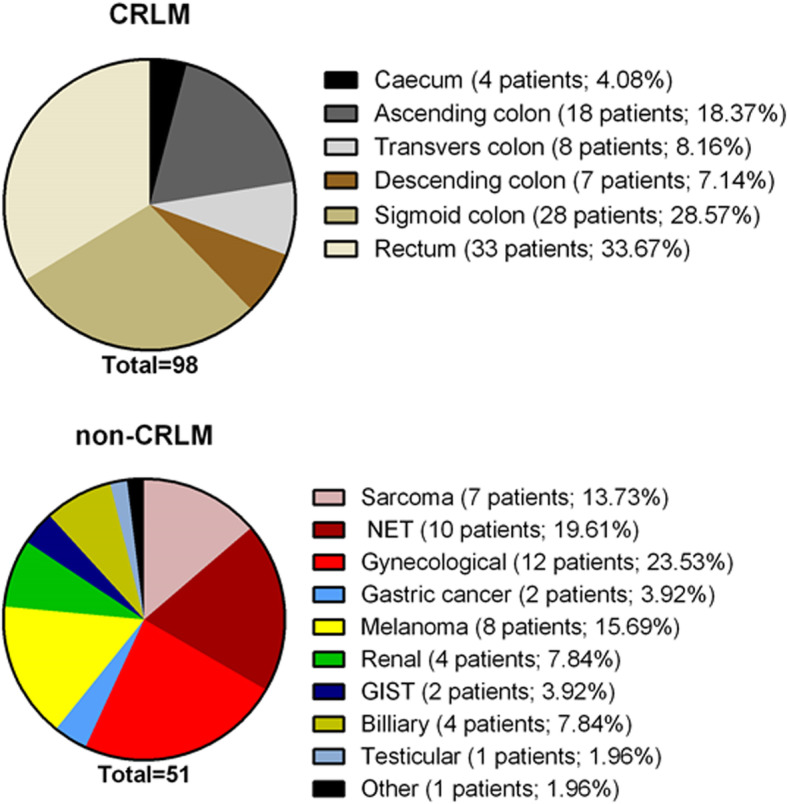
Origin of liver metastases in patients with colorectal liver metastasis and non-colorectal liver metastasis

In the CRLM group, the most common origin of metastases was sigmoid and rectal cancer (52.0%) while in the non-CRLM group gynecological and neuroendocrine tumors (43.1%) (Fig. 1). Metachronous metastases accounted for 86.7% and 94.7% in the CRLM and non-CRLM groups, respectively, *p* < 0.171. The mean number (2.3 ± 2.0 vs 2.4 ± 2.5, *p* = 0.706) and size (3.0 ± 2.7 vs 3.0 ± 3.6 cm, *p* = 0.984) of metastases were not different between the CRLM and non-CRLM groups (Table 2).

**Table 2:** Characteristics of metastases in patients with colorectal liver metastasis and non-colorectal liver metastasis

		CRLM (***n*** = 98)	non-CRLM (***n*** = 51)	***p*** value
Type of surgery	Primary resection; *n* (%)	91 (92.8)	48 (94.1)	0.991
	Secondary resection; *n* (%)	7 (7.2)	3 (5.9)	
Type of metastasis	Metachronous; *n* (%)	85 (86.7)	47 (92.1)	0.171
	Synchronous; *n* (%)	13 (13.3)	4 (7.9)	
Size, cm (mean ± SD)		3.01 ± 0.28	3.0 ± 0.51	0.558

*CRLM* colorectal liver metastases, *non-CRLM* non-colorectal liver metastases, *SD* standard deviation

### Surgery and short-term outcomes

Atypical resection was dominating surgical intervention accounting for 77.8% of all operations. In the CRLM group, 31 (31.6%) patients underwent combined surgical intervention of resection and radiofrequency ablation. There were no differences between the CRLM and non-CRLM groups regarding operation time, intraoperative blood loss, and postoperative blood transfusions (Table 3). Overall postoperative morbidity rate was similar between the CRLM and non-CRLM groups (16.3% vs 9.8%, *p* = 0.402). Major complications by the Clavien-Dindo III-IV rate were similar (10% vs 7.8%, *p* = 0.704) as well. The most common complications were postoperative hematomas, biliomas, and intraabdominal abscesses*.* One (1.0%) patient from CRLM died after surgery, while all patients in the non-CRLM group were successfully discharged from the hospital.

**Table 3:** Perioperative characteristics patients with colorectal liver metastasis and non-colorectal liver metastasis

		CRLM (***n*** = 98)	non-CRLM (***n*** = 51)	***p*** value
Operation length, min (mean ± SD)		198.3 ± 9.2	211.5 ± 12.5	0.353
Blood loss, ml (mean ± SD)		516.8 ± 55.3	572.2 ± 104.6	0.536
Transfusions, units (mean ± SD)		0.56 ± 0.12	0.65 ± 0.22	0.410
Type of liver resection; *n* (%)	Atypical	74	42	0.704
	Right hepatectomy	4	1	
	Left hepatectomy	11	0	
	Segmentectomies	6	5	
	Laparoscopic	3	3	
Postoperative complications by Clavien-Dindo classification; *n* (%)	None	82 (82.7)	46 (90.3)	0.339
	I-II	6 (6.1)	1 (1.9)	
	III-IV	10 (10.2)	4 (7.8)	
	V	1 (1.0)	0 (0)	

*CRLM* colorectal liver metastases, *non-CRLM* non-colorectal liver metastases, *SD* standard deviation

### Long-term outcomes

**Fig. 2:**
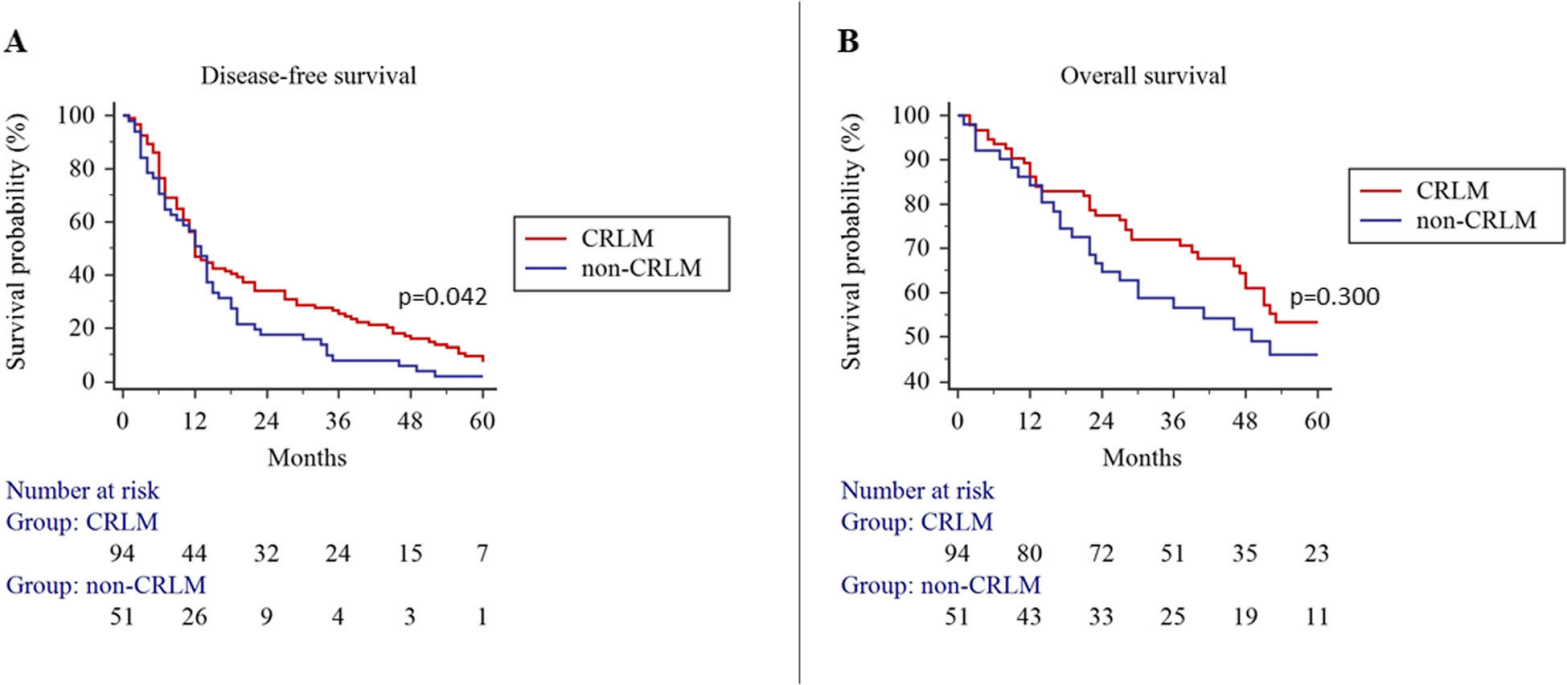
Disease-free and overall survival of patients who underwent liver resection for colorectal liver metastasis and non-colorectal liver metastasis

The median time to follow-up was 38 (Q1;Q3, 22;55) months. Four (2.6%) patients were lost to follow-up. Kaplan-Meier analysis showed higher DFS in the CRLM group with 89.4% vs 76.5% and 64.9% vs 31.4% survival rates at 1 and 3 years, respectively (*p* = 0.042) (Fig. 2a), although OS was not different between the CRLM and non-CRLM groups with 89.4% vs 78.4% and 72.0% vs 46.1% survival rates at 1 and 3 years, respectively (*p* = 0.300) (Fig. 2b).

## Discussion

Liver resection remains the only potentially curative option for patients with liver metastasis. Resection is a well-established option for CRLM; however, its role for non-CRLM remains controversial because of invasiveness and unclear oncological benefits, although continuously improving surgical techniques, better intensive care, and modern chemotherapeutic regimens made liver resection safer and more acceptable option [7]. Thus, patients with non-CRLM may also be considered for radical surgery nowadays.

The present study confirmed similar safety of liver resection for CRLM and non-CRLM. Our findings are consistent with some previous reports suggesting satisfactory short-term outcomes in patients with non-CRLM since the modern and standardized operation techniques are adopted [8–11]. Liver surgery changed in the past 20–30 years after resections became refined, and with an accumulation of evidence, liver parenchyma-sparing techniques, such as atypical resections for metastases, became dominant to preserve liver volume and function [12]. In our cohort, most of all resections were atypical and major resections were limited to cases with multiple lesions in the single lobe. With increasing trends of laparoscopic approach in modern liver surgery, we also incorporated it into our clinical practice, because the laparoscopic approach has been proven to be not inferior to open liver resection in terms of safety and oncological outcomes including surgery for non-CRLM [9, 13–15].

Further, our study showed comparable long-term outcomes after liver resection for CRLM or non-CRLM. Nowadays, the reported 5-year OS rate for CRLM exceeds 50% [7, 16] and similar outcomes were achieved in our study. In contrast, the reported OS rate for non-CRLM varies between 5 and 50% [17–22], and such differences exist because of high heterogeneity for a different type of tumors in this group of patients. In our cohort, the majority of CRLM originated from the left side colon and rectum, which is typically a more common origin for liver metastasis compared to the right colon [2]. Our non-CRLM group was heterogeneous, but most metastases originated from gynecological and neuroendocrine tumors. Such predominance might be associated with a high rate of females in the non-CRLM group compared to the high rate of males in the CRLM group. Females are known to be more susceptible to genitourinary metastasis [23, 24]. Also, some patients in the non-CRLM group had very aggressive cancers, like melanoma, sarcoma, and genitourinary tract cancers. Such tumors are prominent in younger patients [17, 18, 25]; thus, it may lead to age differences between the study groups, where non-CRLM patients were younger. On the other hand, due to the retrospective design of the study, we cannot exclude the selection bias, that elderly patients with non-CRLM were not considered for resection. While the majority of non-CRLM are known for aggressive biological behavior, it was not surprising that DFS in the non-CRLM group was lower, although, despite faster relapses in the non-CRLM group, the comparable OS should encourage surgeons to consider resection for non-CRLM as well.

### Limitations of the study

The present study has several limitations. First, it is a retrospective design study and it might lead to selection bias to perform liver resection for non-CRLM only in very selected cases, although, to our best knowledge, there are no large, prospective, randomized trials evaluating the role of surgery for non-CRLM. Second, the non-CRLM group was heterogeneous by including patients with different types of cancers, although it is important to mention that surgery for non-CRLM remains non-standard; thus, accumulating a sufficient sample size for a homogenous group of one type of metastases is hardly realizable.

Third, the study may be underpowered due to a relatively small number of patients included.

## Conclusions

In this study, we confirmed comparable short- and long-term outcomes after liver resection for CRLM and non-CRLM. Surgical resection should be encouraged as an option in well-selected patients with non-CRLM.

## Data Availability

Data sharing is not applicable to this article as no datasets were generated or analyzed during the current study.

## Abbreviations

CRC: Colorectal cancer
CRLM: Colorectal liver metastasis
DFS: Disease-free survival
OS: Overall survival
